# Transjugular leadless pacemaker implantation after transcatheter tricuspid valve replacement with a biological prosthetic valve: a case report

**DOI:** 10.1093/ehjcr/ytaf609

**Published:** 2025-11-24

**Authors:** Matteo Fortuna, Nicolas Dumonteil, Didier Tchetche, Matthieu Audoubert, Romain Cassagneau

**Affiliations:** Clinique Pasteur, Cardiology Department, Interventional Cardiology, 45 Avenue de Lombez, 31300 Toulouse, France; Clinique Pasteur, Cardiology Department, Interventional Cardiology, 45 Avenue de Lombez, 31300 Toulouse, France; Clinique Pasteur, Cardiology Department, Interventional Cardiology, 45 Avenue de Lombez, 31300 Toulouse, France; Clinique Pasteur, Cardiology Department, Interventional Cardiology, 45 Avenue de Lombez, 31300 Toulouse, France; Clinique Pasteur, Cardiology Department, Interventional Cardiology, 45 Avenue de Lombez, 31300 Toulouse, France

**Keywords:** Leadless pacemaker, Tricuspid valve replacement, Percutaneous valve, Transjugular approach, Complete atrioventricular block, Cardiac implantable electronic devices, Case report

## Abstract

**Background:**

Transcatheter tricuspid valve replacement (TTVR) is an emerging therapy for patients with severe tricuspid regurgitation who are not candidates for surgery. Post-procedural atrioventricular (AV) block may necessitate permanent pacing, but traditional transvenous systems are often contraindicated in the setting of a tricuspid prosthetic valve. Leadless pacemakers represent a valuable alternative, though anatomic changes after valve implantation may challenge standard transfemoral delivery.

**Case summary:**

We report the case of an 82-year-old woman who underwent successful TTVR for severe tricuspid regurgitation. Three days later, she developed complete AV block requiring permanent pacing. Attempts to implant a leadless pacemaker via the right femoral vein failed due to altered tricuspid valve orientation. A transjugular approach enabled effective deployment of the device on the interventricular septum.

**Discussion:**

High-degree AV block is not an infrequent complication occurring after TTVR. Pacing strategies to spare further valve disturbances are mainly represented by single-lead pacing via the coronary sinus and leadless cardiac pacemaker implantation. An individualized approach based on anatomical considerations is essential in the context of complex structural heart interventions.

**Conclusion:**

The transjugular approach can serve as a reliable alternative for leadless pacemaker delivery in patients with tricuspid biological prosthetic valve, offering favourable curvature and access in anatomically challenging cases.

Learning points

- The transjugular approach for leadless pacemaker implantation may provide a more favourable trajectory across the valvular plane in patients with challenging anatomy.
- Leadless pacing after transcatheter tricuspid valve replacement can reduce the risk of prosthesis interference and device-related infections.
- Careful pre-procedural assessment of anatomy and access options is crucial to optimize procedural success and minimize complications.

## Background

Transcatheter tricuspid valve replacement (TTVR) is a growing solution for patients with severe tricuspid regurgitation (TR) who are ineligible for surgery. In some cases, atrioventricular (AV) conduction disturbances post-TTVR require permanent pacing. However, traditional transvenous leads may interfere with the prosthetic valve. Leadless cardiac pacemakers (LCPMs) offer an alternative, but anatomical challenges may preclude standard femoral delivery. This report illustrates how a transjugular approach can overcome these limitations.

## Summary figure

**Figure:**
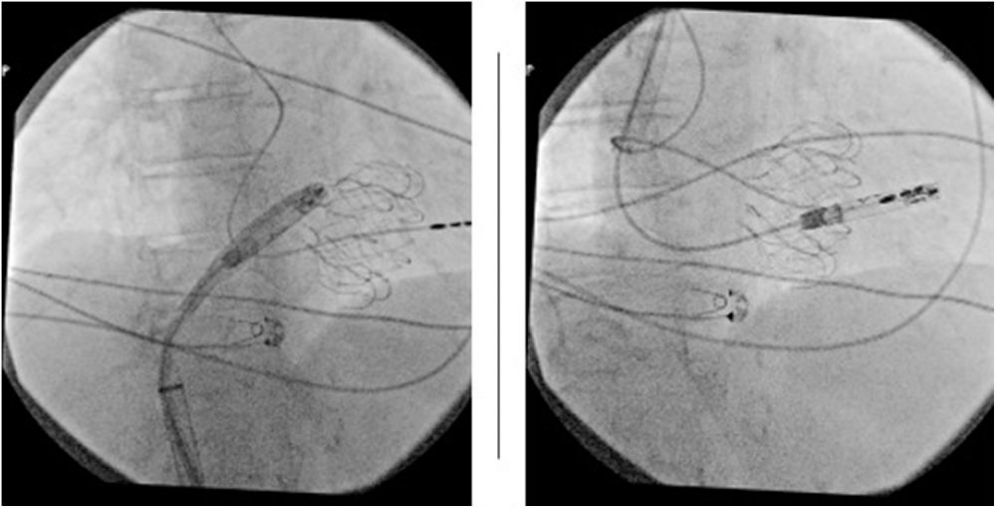
Fluoroscopic images during transcatheter tricuspid valve replacement and subsequent implantation of a leadless pacemaker. Left panel: Deployment of a transcatheter tricuspid valve prosthesis via transfemoral venous access. Right panel: Final positioning of the leadless pacemaker delivered through a transjugular approach, successfully anchored within the right ventricular septum, with the tricuspid prosthesis *in situ*. This combined strategy was adopted following the failure of the femoral attempt due to interference with the newly implanted prosthetic valve.

## Case summary

An 82-year-old woman was referred for elective percutaneous tricuspid valve replacement due to symptomatic severe secondary TR. The clinical status was characterized by the presence of a prominent right-sided heart failure and New York Heart Association class III, with peripheral oedemas and organomegaly with rise in liver enzymes and alteration of the coagulation panel. Her medical history includes permanent atrial fibrillation, ischaemic stroke without neurological sequels, and a myocardial infarction treated with thrombolytics. Pre-procedural echocardiography revealed preserved left ventricular function and moderate mitral and aortic regurgitation. Baseline electrocardiogram (ECG) showed atrial fibrillation with a bifascicular block (right bundle branch block and left anterior fascicular block).

A 52 mm tricuspid biological prosthetic valve (Evoque™, Edwards Lifesciences) was successfully implanted via a transfemoral approach. The patient was extubated immediately post-procedure with excellent haemodynamic and echocardiographic results, underlined by a residual mild TR with a good contractile function of the right ventricle without the need for inotropic support.

On postoperative Day 3, continuous telemetry monitoring revealed sudden-onset complete AV block with hypotension, prompting urgent temporary pacing achieved through a transjugular approach with an active-fixation lead and having the external generator anchored to the cervical region.

The following day, confirming the absence of a valid intrinsic conduction and the presence of a low escaping rhythm that did not guarantee a valid haemodynamic support, an indication for a permanent pacemaker implantation (PPI) was confirmed. Considering the presence of a tricuspid biological prosthetic valve, a leadless pacemaker (Aveir VR™, Abbott) was selected to avoid permanent transvalvular leads.

**Figure 1:**
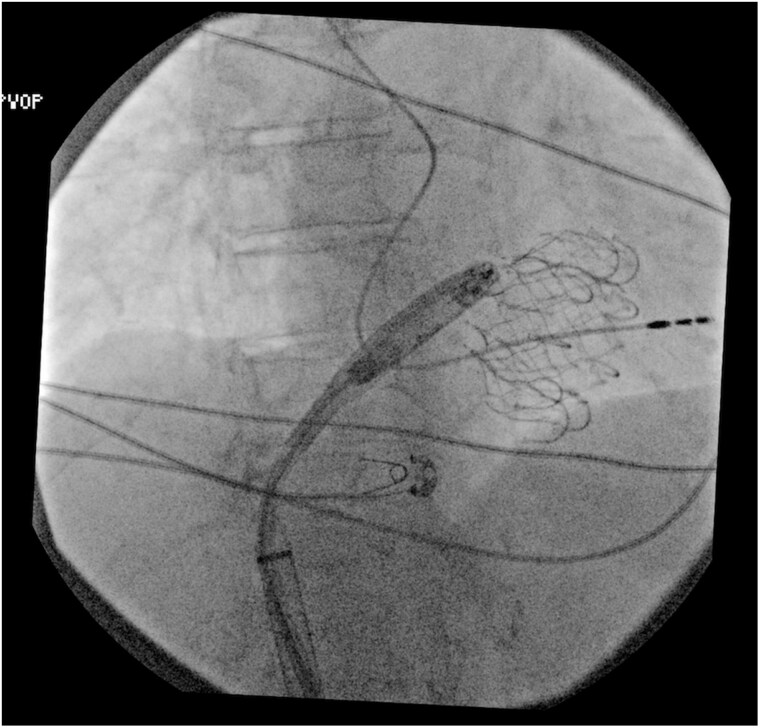
Fluoroscopic right anterior oblique view showing failed transfemoral attempt: delivery catheter unable to navigate the prosthetic tricuspid annulus due to its superior-lateral displacement.

**Figure 2:**
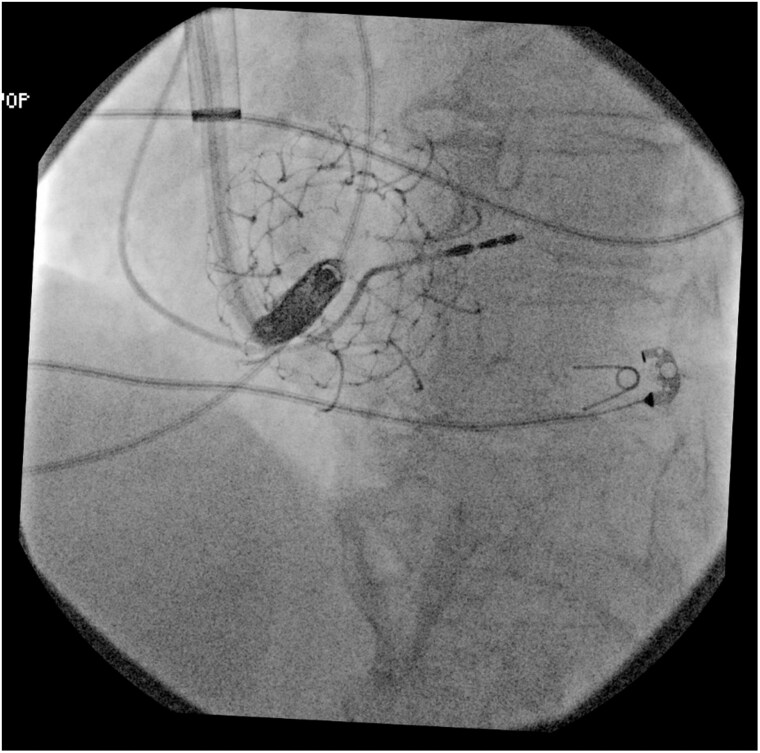
Left anterior oblique view during transjugular implantation: the delivery system achieves optimal curvature and perpendicular alignment to the septum, allowing device deployment.

**Figure 3:**
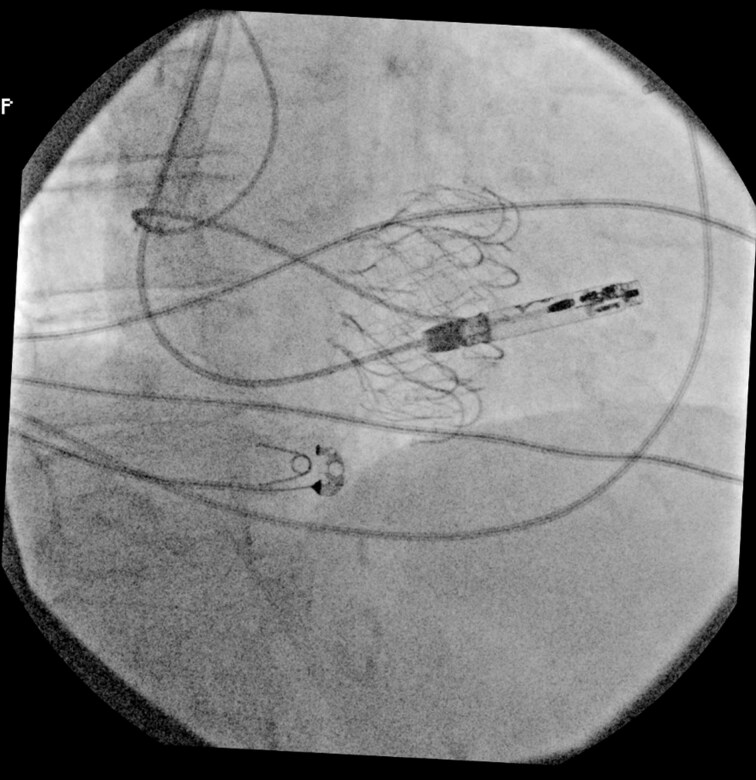
Right anterior oblique view during transjugular implantation showing appropriate leadless pacemaker position in the mid-septum.

**Figure 4:**
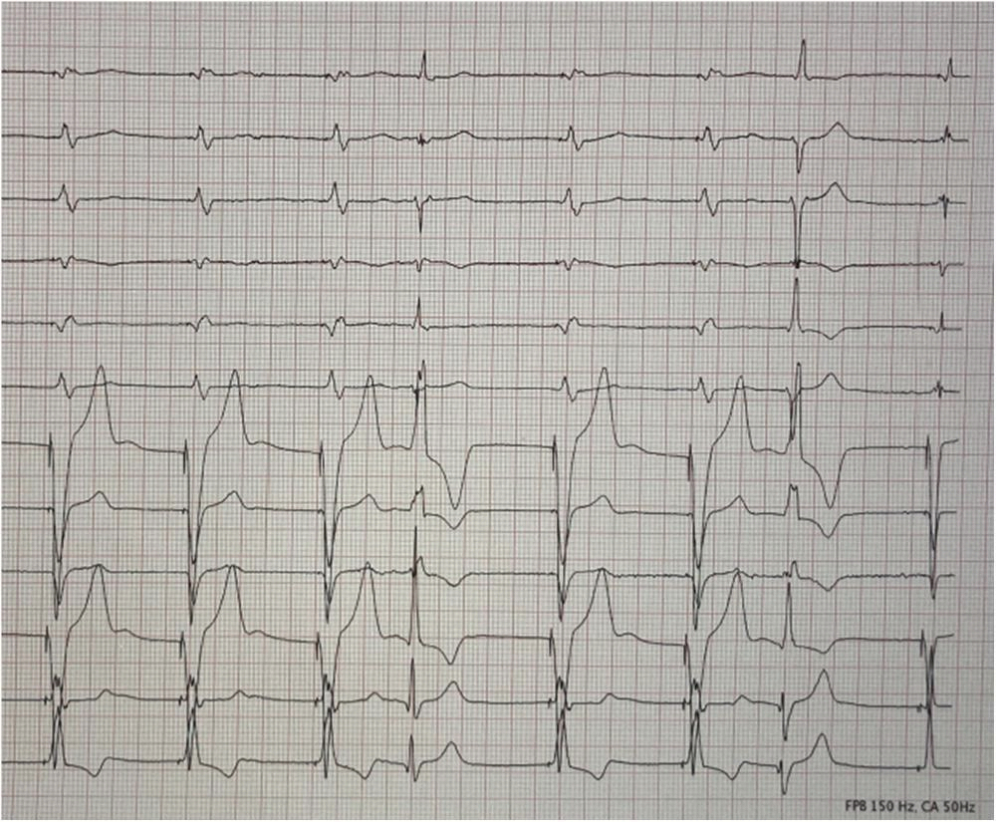
Twelve-lead ECG on the first postoperative day showing appropriate septal ventricular pacing. Two intrinsic beats with bifascicular block are also visible.

Initial conventional transfemoral attempts failed due to prosthetic valve-induced displacement of the tricuspid annulus in a more superior and lateral position (*Figure 1*). This anatomical distortion prevented the transfemoral delivery system from achieving the necessary curvature to cross the valvular plane without interfering with the newly implanted prosthetic valve. Consequently, an ultrasound-guided transjugular approach was undertaken using the dedicated Abbott transjugular delivery sheath. This route provided enhanced manoeuvrability and curvature within the right atrium, in particular the superior offset angle was larger and provided also the possibility of ‘leaning’ the delivery sheath against the anterior and lateral walls of the right atrium, thus favouring a steeper angle for crossing the prosthetic valve without pushing the system too close to the edges of the valvular plane. This approach enabled successful deployment of the leadless pacemaker on the interventricular mid-septum (*Figures 2* and *3*). The puncture site was closed using a simple figure-of-8 suture and a 5-min manual compression. Furthermore, the supine posture with slight head elevation reduces jugular venous pressure and thus facilitates haemostasis at the puncture site. No early nor late vascular complications occurred, and immediate post-operative ambulation was achieved. The device was programmed in VVI mode at a lower rate limit of 55 b.p.m. A 12-lead ECG obtained on the first post-operative day confirmed appropriate septal ventricular pacing (*Figure 4*).

A follow-up chest X-ray confirmed proper positioning, and the patient was discharged 2 days later in stable condition, with optimal device function. At subsequent outpatient follow-ups, appropriate sensing and pacing thresholds were assessed with partial recovery of intrinsic conduction and a percentage of ventricular pacing ranging from 35% to 50%.

## Discussion

As recently showed in the TRIPLACE registry,^1^ the incidence of new high-degree AV block (HAVB) after a TTVR procedure is 13.5% at 1-month follow-up in a naïve population, having HAVB occurrence at a median of 3 days after the index procedure. Left bundle branch block (LBBB) or fascicular block—anterior as well as posterior—has been recognized as independently associated with a higher risk of post-operative HAVB. In this selected population, the main choice (45.5%) for PPI was represented by LCPM. The aetiology of these conduction disturbances is likely multifactorial. Nodal tissue injury may result from mechanical interaction with the prosthesis at the tricuspid annulus, potentially compounded by local oedema or ischaemia.

As worsening TR following cardiac implanted electronic device (CIED) implantation ranges from 10% to 39% according to the latest registries,^2^ it seems of the utmost importance to avoid as far as possible to endanger the prosthetic valve or its function. Of course, this need must meet the ‘electrophysiological’ needs for a safe, reliable, and possibly long-lasting pacing device.

Out of the existing possibilities discussed in the recent EHRA-EAPCI consensus paper,^3^ we will consider mostly two options: coronary sinus (CS) pacing and LCPM implantation. While CS pacing offers the undeniable benefit of avoiding valve—or prosthetic valve—disturbances, it appears more challenging to optimize the electrophysiological parameters; furthermore, phrenic stimulation, lead displacement, and chronic high thresholds represent potential challenges.

Leadless cardiac pacemaker on the other hand offers the advantage of an endocardial stimulation, more reliable chronic thresholds, low rates of valve disturbances, and the absence of intravascular leads, which may complicate the treatment of concurrent infective endocarditis (IE) in the context of a tricuspid prosthetic valve.

Having weighted the potentials benefits and downsides of the two available techniques, LCPM seems to us the preferable choice for our patient.

The complexity of LCPM implantation after TTVR is not solely represented by the priority of preserving the prosthetic valve function but also by the presence of the prosthetic valve itself, which displaces the tricuspid annulus in the context of an already known challenging right atrium anatomy.

As described in the work of Saleem-Talib *et al*.,^4^ transjugular approach is a feasible alternative for the LCPM implantation to the conventional transfemoral approach; moreover, an enhanced device manoeuvrability allowing the operator to tailor the pacing site to reach more physiological pacing parameters (evidenced by a narrower QRS) and the possibility of immediate post-operative ambulation seem compelling advantages.

If ultrasound-guided venous puncture is properly performed, the rate of vascular complications with transjugular access is comparable to that observed with ultrasound-guided transfemoral access, as remarked by the absence of serious vascular complications in the series of 82 transjugular LCPM implantation in the work of Saleem-Talib *et al*.

Furthermore, the work of Soejima *et al*.^5^ underlines the importance of acknowledging that not all hearts are the same, and the old saying ‘one size does not fit all’ sticks also for CIED: in a mixed cohort of American and Japanese patients needing PPI with LCPM, heart dimensions were derived from CT scans, and most of the Japanese cohort demonstrated challenging anatomical features that prevented easy transfemoral LCPM delivery. The curve from the inferior and superior vena cava (IVC and SVC, respectively) to the right atrium, the angle between the IVC–SVC axe and the tricuspid valve plane, the heart size, and the angle to reach the interventricular septum are the dimensions that were held predictors for a tortuous transfemoral route hinting that in selected populations, a transjugular approach may be considered in the first place.

In conclusion, we reckon that a tailored approach should be considered in view of increasing percutaneous interventions on the tricuspid valve.

Our case, alongside other reported case reports,^6^ supports the use of transjugular route for LCPM implantation.

## Conclusion

This case demonstrates the feasibility of transjugular leadless pacemaker implantation in a patient with a transcatheter tricuspid biological prosthetic valve. In a scenario of altered orientation of the tricuspid annulus secondary to TTVR, the curvature offered by this approach has been more favourable. Awareness of alternative access routes and anatomical considerations is crucial to ensure optimal outcomes in the era of structural valve interventions.

## Lead author biography


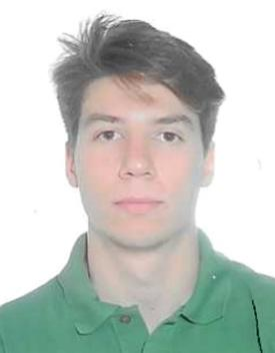


Matteo Fortuna is a cardiologist and electrophysiology fellow at Clinique Pasteur in Toulouse, France. He previously trained and completed his cardiology residency at the University of Milano-Bicocca. Dr Fortuna has international clinical and research experience in Vienna, New Delhi, and Maastricht. His current focus is on complex arrhythmia management, leadless pacing, and innovative device-based therapies. He combines a strong clinical background with a passion for research in the field of cardiac electrophysiology.

**Consent:** Written informed consent was obtained from the patient, and all efforts have been made to anonymize personal information and ensure patient confidentiality.

## Data Availability

Data sharing is not applicable to this article as no new datasets were generated or analysed during the current study.

## References

[1] Scotti A, Puri R, Sturla M, Zahr F, Boone R, Kodali S, et al Incidence, predictors, and management of conduction disturbances after transcatheter tricuspid valve replacement. JACC Cardiovasc Interv 2025;18:1789–1799. 40637674 10.1016/j.jcin.2025.05.029

[2] Hahn Rebecca T . Tricuspid regurgitation. N Engl J Med 2023;388:1876–1891. 37195943 10.1056/NEJMra2216709

[3] Deharo JC, Dreyfus J, Bongiorni MG, Burri H, Defaye P, Glikson M, et al Management of patients with transvalvular right ventricular leads undergoing transcatheter tricuspid valve interventions: a scientific statement of the European Heart Rhythm Association and the European Association of Percutaneous Cardiovascular Interventions of the ESC endorsed by the Heart Rhythm Society, the Asian Pacific Heart Rhythm Society and the Canadian Heart Rhythm Society. Europace 2025;27:euaf061. 41243769 10.4244/EIJ-JAA-202501 PMC12599824

[4] Saleem-Talib S, Van Driel VJ, Nikolic T, Van Wessel H, Louman H, Borleffs CJW, et al The jugular approach for leadless pacing: a novel and safe alternative. Pacing Clin Electrophysiol 2022;45:1248–1254. 36031774 10.1111/pace.14587

[5] Soejima K, Hilpisch K, Samec ML, Temple RL, Bonner MD. Jugular approach for the transcatheter pacemaker implant―better access for smaller hearts? Circ J 2024;88:1127–1134. 38658350 10.1253/circj.CJ-24-0083

[6] Hale BW, Bradley DJ, Zampi JD, Whiteside W, Cunnane R. First-in-human combined transcatheter tricuspid valve implantation with leadless VDD pacemaker via left internal jugular approach. HeartRhythm Case Rep 2021;8:155–159. 35492842 10.1016/j.hrcr.2021.11.020 PMC9039545

